# Medicinal Effects of Saleratus

**Published:** 1855-03

**Authors:** 


### *Medicinal Effects of Saleratus.*

Our attention has been directed to a paper in the *Boston Medical and Surgical Journal,* by Dr. W. A. Alcott, entitled, "Mortality among Children," wherein the author attributes the great mortality of children under five years of age, amounting to three fifths of all who die, principally to the use of saleratus in bread. This bold assertion is made without even an attempt to substantiate it by facts, other than the statement that Orfila places it in the list of irritant poisons. This, undoubtedly, is true, but the same list contains nearly all the saline substances that are in general use, common salt not excepted, several cases being mentioned by Orfila where death has resulted from the excessive use of salt itself.

Criticism upon such idle statements would not justify the use of the space required in a medical journal, if the readers of the original articles were medical men alone; but the various periodicals and newspapers of the day greedily copy remarks of this nature, since it cannot but excite a lively astonishment in the minds of their readers that they have survived the frequent use of such deadly poisons. It is needless to say that nineteen-twentieths of those who use this substance are perfectly ignorant of its nature and properties, merely knowing that saleratus is saleratus, and are quite as ready to believe it to be a poison as they would tartar emetic or blue vitriol. It is, therefore, due to the public that they should know whether they have been needlessly alarmed by the vagaries of a visionary, or whether they have indeed for years been slowly poisoning themselves with this drug.

Before entering upon any investigation into the properties of this substance, it is necessary to give a few passages from Dr. Alcott's paper. He observes:

"In regard to the causes of this fearful and fearfully increasing infantile mortality—for there are doubtless more causes than one—I have something to say, suggested by the study of the subject for thirty years or more. And though I lay no claim to infallibility, I do greatly desire to be heard.

"My first suspicion rests on the too free use of alkalies among us. I say 'the too free use,' because, although I should not be likely to encourage their dietetic use, in any quantity, or in any circumstances of *health*, yet there is certainly a wide difference between excess and moderation. It is one thing to use just so much saleratus as shall be neutralized by the acetic acid it meets with, so as to leave no residuum but a little acetate of potash, and quite another to use the alkali so freely that a portion of it remains in the stomach and intestines unneutralized. Yet the latter is an every-day occurrence. Our children, generally, have their first passages in a state of sub-inflammation, from this and other kindred causes; and though the use of mild acids, especially those of fruits, may do something to soften or mitigate the condition, is it any wonder that bowel complaints, in these circumstances, become very severe and unmanageable? Is it any wonder that two-fifths, and in summer three-fifths of all who are born, die under five years of age?"

"My deliberate conviction is, that the families of twenty millions of people in our United States population—amounting to about four millions—use the average quantity of five pounds of this alkali yearly—or one pound to each individual. This is an aggregate of twenty millions of pounds. How much of this goes into the alimentary canal and courses its devious way without meeting with any free acid or other substance calling into play new affinities, cannot easily be told. In these days of excess in its use, I fear one-half. But to be safe, I will place it at one-fourth. Is it so, then, that the lining membranes of our people—

our children among them—must be irritated yearly by 5,000,000 pounds of uncombined, unneutralized saleratus? The very thought is enough to make one shudder!

"From ten to twenty grains of this substance is sometimes put down in our medical dictionaries as a dose. Place it at thirty. Do we swallow 960,000,000 doses of medicine a year, in this careless, uncalled-for manner? What effect can medicine be likely to have, when given in an emergency, to children who have been irritated day after day, and year after year, in this way? I have said irritated—not poisoned. Yet Orfila, I find, calls saleratus an irritating poison, and gives us a long list of its terrible symptoms."

Dr. Alcott forgets that at least one half of the number of deaths here cited must have been of children under one year of age, most of whom have never eaten bread of any sort, *with* or *without* saleratus; it consequently cannot have been the exciting cause in these instances.

The substances sold under the name of saleratus, may be divided into three varieties, viz.: potash saleratus, soda saleratus and bi-carbonate of soda. The latter is met with generally in a state of almost absolute purity. The other two consist of variable mixtures of carbonate and bi-carbonates of the above named alkalies, contaminated with the impurities existing in the materials from which they were prepared, *i. e.* pearl ash and soda ash.

Soda saleratus contains, generally, a small percentage of sulphate of soda, and a large proportion of common salt. Potash saleratus contains all the saline ingredients capable of existing in the infusion of wood ashes, from which it is derived, principally sulphates, chlorides and phosphates of the alkalies, and traces of the corresponding lime salts. It is unnecessary to take their medicinal action into account, being contained in too small amount to exert any appreciable action upon the animal economy. We need, therefore, only direct our attention to the effect of the carbonates and bi-carbonates of potassa and of soda, bearing in mind, however, that in all cases where saleratus is used judiciously, that is, in combination with an acid in the ratio of their respective equivalents, or the acid slightly in excess, the salt present in the bread must necessarily be a compound of the acid used; consequently, the acetate, lactate of soda or of potassa, in place of the carbonates of the same basis; the acetic, lactic and tartaric acid being almost exclusively the only ones ever present, whether intentionally added, or present in the form of sour flour or milk. All of these salts above named are *very* gentle purgatives; most, if not all, of them possessing less action, and requiring to be administered in a larger dose then even common salt itself, (from ½ oz. to 1 oz.) No injurious effects can possibly, therefore, arise from their constant use in the small quantities here occurring.

From 30 to 40 grains of saleratus is more than sufficient for raising bread made from two pounds of flour, or enough for a meal of twelve persons. Taking the larger amount, 40 grains, each person will consume about 3½ grains at once. Supposing, from carelessness or ignorance in the use of it, that the whole amount should remain unsaturated, and consequently remain in the form of carbonate or bi-carbonate, could the above quantity produce any bad effects?

It is well known that the nature of the action of most saline substances, depends much upon the state of concentration of the solution in which it is exhibited. Golding Bird* says:

"When, therefore, saline substances, especially, are intended to be absorbed and ultimately to reach the kidneys, it is necessary that the density of their solutions should be much below 1.028; the proportion of solids dissolved in the aqueous vehicles prescribed being always less than five per cent. Daily experience in the employment of remedies will show the importance of this law in a therapeutical sense. Thus, a tolerably strong solution of the tartrate, or acetate of potass, will altogether escape the absorbents; indeed, so far from being imbibed by the capillaries, it will actually excite an exudation of water from these vessels in the stomach and small intestines, thus becoming diluted by exosmosis, and a sensation of thirst is excited, by which the patient is compelled to drink for the purpose of supplying the water removed from the blood by exudation. In strong solutions, the salts alluded to, stimulate the bowels and purge. They are, moreover, said to act as *hydragogue* purgatives, producing watery motions—a fact also capable of ready explanation on physical laws; exudation of water from the exhalents (capillaries) occurring, on account of the density of the saline solution traversing the intestines, just as exosmosis was produced in the experiment of the tube of water immersed in syrup. We can hence readily perceive why half an ounce of acetate or tartrate of potass will purge, and a scruple of either, excite diuresis."

Dr. Parkes, in his valuable paper upon the action of liquor potassæ in health and in disease, remarks:

* "Bird on Urinary Deposits," 2d American Edition.

"The bicarbonate of potash, therefore, had the following action: it passed off very rapidly and in part as carbonate or bicarbonate, with the urine, and therefore, increased the amount of the soluble salts; it augmented, also, the water by 50 oz., (the calculation was made for twenty-four hours,) the organic solids by 264 grains, the sulphuric acid by 7 grains, and the sulphur by more than 5 grains.

"The action of the bicarbonate of potash appears so far to differ from that of the liquor of potassæ that some considerable portion passes off unchanged in the urine; whenever this is the case, the urine is rendered alkaline. This passage occurs with such rapidity, that if it is wished to keep up the increased alkalinity of the blood, the salt must be given very frequently, (every hour or so,) if given only every four hours, in less than two it is excreted, so that for two hours the blood is unacted upon."

In the above experiment ʒiv. were administered in the space of 13 hours, being about 60 times the quantity that would probably be taken in bread during the same time. It must now be evident that no *constitutional* injury could arise from the use of alkalies in small quantities. With regard to the action exerted by the irritant properties of the alkalies upon the coats of the stomach and intestines, Dr. Parkes' experiments with liquor potassæ are most conclusive. We extract his results at length.

"If this remedy be taken soon after meals, its action is that of an antacid. It combines with hydrochloric or with lactic acid, and then, doubtless, passes into the circulation. What appreciable effect it now produces is not indicated in the tables above given, but it does not increase either the water, solids, or sulphuric acid of the urine. If the liquor potassæ be taken into an empty stomach, it passes unneutralized into the circulation, and probably through the veins; in so doing it must produce an effect on the walls of the capillaries and small veins; but the extent of this cannot be known. As much as ʒij have been taken with only 4 oz. of water, without causing epigastric pain or uneasiness (although it produced considerable temporary scalding of the mouth and throat), and without apparently producing any local effects in the stomach. In, usually, from thirty to ninety minutes after its entrance into the circulation, an increased flow of slightly acid urine occurs, which contains the whole of the potash, organic matter differing considerably from that of ordinary urine, and a relatively large proportion of sulphuric acid; the phosphoric acid and the chlorine are less changed. Perhaps an organic acid (not uric, and probably not hippuric) is also present.

"The explanation of these facts is, that an albuminous compound, either in the blood itself, or in the textures, has become oxidized; its sulphur, under the form of sulphuric acid, has united with potash, and, with possibly the changed protein-compound, is poured out from the kidneys. This oxidizing effect of the liquor potassæ is no doubt assisted by exercise, and by copious draughts of water; but in the above experiments, exercise and fluid were abstained from, in order not to complicate the results. The amount of albumen or fibrin destroyed by one drachm of liquor potassæ cannot be considerable, but if the potash were continued in large quantities, oxidation could probably be pushed to any amount. The nitrate and acetate of potash did not in a *healthy system* have the same effects.

"After the increased flow of urine, the quantity passed per hour falls slightly below the standard. It appears to resume its ordinary composition, but its exact condition at this period has not been determined. Some observations on urine in disease would lead me to infer that the uric acid will be found to be increased.

"Such were the effects of liquor potassæ on the urine. The effect produced on other excretions was not obvious. The skin and the intestines appeared quite unaffected, and as all the potash was found in the urine, the reason of this is easily understood. In most of the experiments there were no subjective symptoms of any kind. On two occasions, there was rather sharp frontal headache, languor, depression, slight lumbar pain, and aching of the legs, after the large flow of urine. On the night of the 15th, when the flow of urine, which was proceeding at the rate of ℥iss per hour, was augmented in two and a half hours by ℥xiv, and no fluid was supplied to the system, the pulse became perceptibly small (almost thready) and slow; it remained equal and regular—there was no thirst, no shivering, and no nausea; the skin was dry and warm. In six hours the pulse had quite regained its force and frequency, and the other symptoms had disappeared without any fluid having been taken.

"After the experiments were concluded, the general health did not appear impaired; it was, if anything, better than usual."

When we recollect that liquor potassæ, (solution of hydrate of potassa,) in its action upon animal tissues is, without exception, the most powerful chemical agent that we possess, rapidly distroying and dissolving almost all organic structures *out of the body* with which it comes in contact, we can judge how slight must be the chemical changes produced upon the mucous membranes by the bi-carbonates, being as they are, almost devoid of all alkaline reaction.

If the ill consequences resulting from careless and bad cooking were properly estimated, it would be found that much disease might be traced to sour or badly fermented bread. Dr. Drake, in the first part of his valuable work on the "Diseases of the Valley of America," attributes many of the prevailing ailments to the following, among other causes.

"When the dough for leaven, by the excess of panary fermentation, has been charged with acetic acid, that product is not in general neutralized by carbonate of potash or of soda, but the bread is eaten sour."

It is unnecessary to deduce further proofs of the slight effect produced by the salts above mentioned. There can be but little doubt that the dangerous properties attributed to saleratus by some persons, exist entirely in their imagination. C.

